# Impact of age on short-term outcomes after pancreaticoduodenectomy: A retrospective case-control study of 260 patients

**DOI:** 10.3389/fsurg.2023.1031409

**Published:** 2023-03-30

**Authors:** Zhirong Zhao, Shibo Zhou, Yaping Tang, Lichen Zhou, Hua Ji, Zheng Tang, Ruiwu Dai

**Affiliations:** ^1^ Hospital of Southwest Jiaotong University, The General Hospital of Western Theater Command, Chengdu, China; ^2^ General Surgery Center, General Hospital of Western Theater Command, Chengdu, China; ^3^ College of Clinical Medicine Southwest Medical University, Luzhou, China; ^4^ Pancreatic Injury and Repair Key Laboratory of Sichuan Province, General Hospital of Western Theater Command, Chengdu, China

**Keywords:** pancreaticoduodenectomy, age groups, elderly patients, Clavien-Dindo score, POPF

## Abstract

**Background:**

Although the increase of perioperative complications in the elderly undergoing pancreaticoduodenectomy (PD) surgery has been recognized, the definition of the “old patient” of PD in the studies is different and there is no accepted cut-off value at present.

**Methods:**

279 consecutive patients who have undergone PD in our center between January 2012 and May 2020 were analyzed. Demographic features, clinical-pathological data and short-term outcomes were collected. The patients were divided into two groups, and the cut-off value (62.5 years) is picked based on the highest Youden Index. Primary endpoints were perioperative morbidity and mortality, and complications were classified according to the Clavien-Dindo Score.

**Results:**

A total of 260 patients with PD were included in this study. Postoperative pathology confirmed pancreatic tumors in 62 patients, bile duct tumor in 105, duodenal tumor in 90, and others in 3. Age (OR = 1.09, *P* < 0.01), and albumin (OR = 0.34, *P* < 0.05) were significantly correlated with postoperative Clavien-Dindo Score ≥3b. There were 173 (66.5%) patients in the younger group (<62.5 years) and 87 (33.5%) in the elderly group (≥62.5 years). Significant difference between two groups was demonstrated for Clavien-Dindo Score ≥3b (*P *< 0.01), postoperative pancreatic fistula (*P *< 0.05), and perioperative deceases (*P *< 0.05).

**Conclusions:**

Age and albumin were significantly correlated with postoperative Clavien-Dindo Score ≥3b, and there was no significant difference in predicting the grade of Clavien-Dindo Score. The cut-off value of elderly patients with PD was 62.5 years old and there were useful in predicting Clavien-Dindo Score ≥3b, pancreatic fistula, and perioperative death.

## 1. Introduction

As important basic data in medicine, age is directly related to decision when surgeons consider the operation. A patient's age can affect the complication and prognosis after surgery, and the patient of elderly often suggests poor surgical outcomes (1, 2). In 2019, 617 million people are 65 years old or older; by 2050, the number will reach 1.6 billion—nearly 20% of the world's population (3). Inevitably, surgeons have to be confronted with the surgical choices of more elderly patients.

Pancreaticoduodenectomy (PD) is still burdened by high rates of complication and mortality (4). The poor outcomes of PD is primarily owing to the highly malignant periampullary neoplasms and the great trauma of operation on human body (5). Considering the potentially higher risks of age-related complication and mortality, the decision to submit elderly to PD would be challenging (6, 7). Early identification of elderly patients, combination of perioperative vigilance and proper treatment may help to reduce the incidence of serious complications and postoperative death. However, how to define the elderly in PD remains controversial. At present, the age group of patients with PD surgery is based on the aging population (8). The criteria for the elderly in some research were the aging criteria of the World Health Organization, such as 65 and 75 years old (9). In another study, regarding the age of 70 as the standard of the elderly is a subjective judgment (9, 10). There is a lack of calculation of the cut-off value of the age of patients with PD (11).

In the present study, we aimed to analyze the effect of age on short-term outcomes in patients undergoing PD and to determine the criteria for the elderly for PD. In particular, it was desired to determine whether differences exist between young and elderly people in postoperative complications and mortality. By calculating the cut-off value of age for PD, the short-term prognosis evaluation effect was calculated, which is helpful for the judgment of PD.

## 2. Methods

### 2.1. Patients

**Figure 1:**
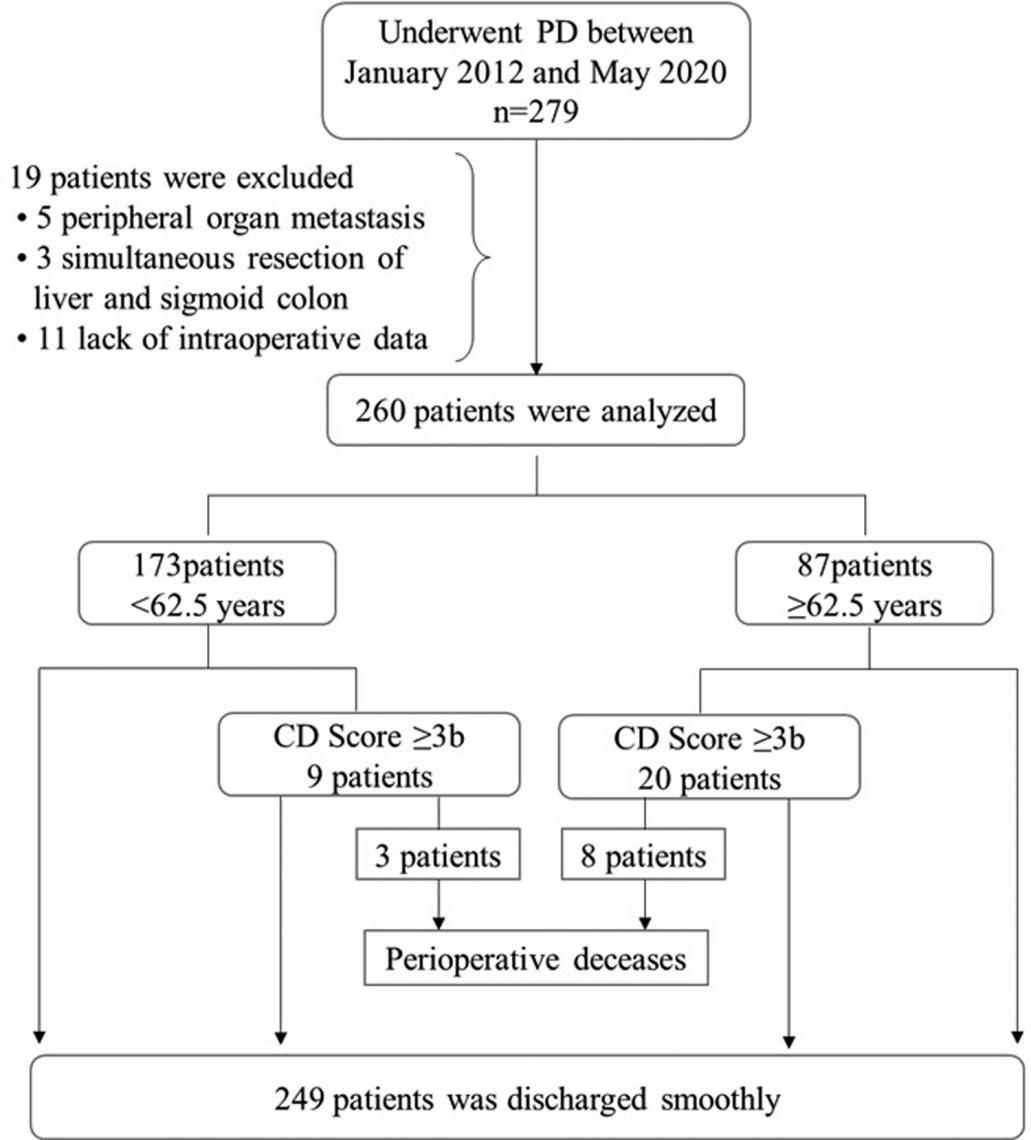
Inclusion, exclusion and outcomes of patients. CD Score, Clavien-Dindo Score; PD, Pancreaticoduodenectomy.

The medical records of all patients who underwent PD in our center between January 2012 and May 2020 were analyzed (*n* = 279). 19 subjects were excluded because of lack of data or other concomitant procedures (Figure 1). The same surgical team performed all PD using the same procedure, and pathological examination was performed after operation. Age, sex, body mass index (BMI), hypertension, diabetes, hemoglobin, total bilirubin, albumin, complication, re-operation, perioperative mortality (30-day or in-hospital), hospital stay, cost, readmission (90 days of initial discharge), and tumor pathological characteristics were recorded for each participant. The study was approved by the ethics committee of the General Hospital of Western Theater Command and was conducted in accordance with the principles of the Helsinki declaration. The ethical approval number is 2021EC2-26.

### 2.2. Surgical technique and postoperative management

The PD was performed by the fixed team, which was composed of five experienced pancreatic surgeons, including at least two attending surgeons. All patients were performed standard Whipple operation for side-to-side gastrojejunal anastomosis, end-to-side biliary jejunal anastomosis and end-to-side pancreatic jejunal anastomosis. The extraperitoneal drainage tube was placed behind the cholangiojejunostomy and pancreaticojejunostomy. Antibiotics were used prophylactically during operation and octreotide was used routinely after operation. The abdominal drainage tube was removed if the amylase level of drainage fluid was normal for three consecutive times after operation.

### 2.3. Classification of surgical complications

Grade I: Any deviation from the normal postoperative course without the need for pharmacological treatment orsurgical, endoscopic, and radiological interventions.

Grade Il: Requiring pharmacological treatment with drugs other than such allowed for grade I complicationsBlood transfusions and total parenteral nutrition are also included.

Grade IIl: Requiring surgical,endoscopic or radiological intervention.

Grade IIla: Intervention not under general anesthesia.

Grade IIlb: Intervention under general anesthesia.

Grade IV: Life-threatening complication requiring IC/ICU management.

Grade IVa: Single organ dysfunction (including dialysis).

Grade IVb: Multiorgan dysfunction.

Grade V: Death of a patient.

### 2.4. Definition of complications

Postoperative complications were defined as follows, and Clavien-Dindo Score was used to evaluate the postoperative complications (12–15):

Biliary leak: bilious drainage from intraoperatively placed drains or radiographically proven fluid collection requiring percutaneous drainage and demonstrating elevated bilirubin levels.

Postoperative pancreatic fistula (POPF): POPF is now redefined as a drain output of any measurable volume of fluid with an amylase level >3 times the upper limit of institutional normal serum amylase activity three days later after surgery, associated with a clinically relevant development/condition related directly to the postoperative pancreatic fistula.

Chyle leak: Chyle leak was defined as output of milky-colored fluid from a drain, drain site, or wound on or after postoperative day 3, with a triglyceride content ≥110 mg/dl (≥1.2 mmol/L).

Postoperative bleeding: Blood loss proved by various ways, such as hematemesis, hematochezia or melena.

Intraperitoneal infection: Intraperitoneal fluid obtained from percutaneous drainage with culture-proven bacterial organisms.

Delayed gastric emptying: radiological evidence of delayed gastric emptying or post-operative nasogastric tube decompression longer than 2 weeks.

Pulmonary infection: fever, leukocytosis, sputum with leukocytosis, on culture of sputum demonstrating a pathogen, and chest radiograph demonstrating focal infiltrates.

Incision dehiscence: any disruption of the skin closure with subcutaneous tissue exposure or a wound opening that required packing.

Pleural Effusion or Ascites: evidence of fluid collection on postoperative imaging.

Wound infection: Wound infection is defined as findings including purulent discharge, localized swelling, redness/heat, delayed wound healing, or positive wound culture results.

### 2.5. Statistical analysis

We report the categorical variables as absolute value and percentage value, continuous variables as average ± standard deviation, and asymmetrical distribution (Skewed distribution) continuous variables are presented as median [interquartile range]. A *P*-Value <0.05 was considered statistically significant. A univariate logistic regression analysis was then performed to identify preoperative variables associated specifically with perioperative complication (Clavien-Dindo Score ≥3b). Variables tested in this analysis included age, sex, BMI, comorbidities (hypertension, diabetes), and laboratory value (hemoglobin, total bilirubin, albumin). Multivariable analyses were carried out in variables with significant differences in univariate logistic analysis. The strength of the association between postoperative complications and age was evaluated by calculating the area under the respective receiver operating characteristic (ROC) curves. This is defined as Y = sensitivity + specificity −1 and ranges from 0 to 1. The dimensional cut-off maximizing the balance between sensitivity and specificity in predicting greater postoperative complications was identified through calculation of the Youden Index, and a cut-off value is picked based on the highest Youden Index. According to the cut-off value, the age groups were divided, and the short-term outcomes which included complications, perioperative deceases, readmission, hospital stay (length of stay after operation), and cost were analyzed. Comparison between the frequencies of the categorical variables (complications, perioperative deceases, readmission,) was assessed using the Chi-square test. The Mann-Whitney U test was used to compare the differences between hospital stay, cost, and in pairs of group. The variables which significant differences in univariate logistic with Clavien-Dindo score were analyzed by ordinal logistic analysis. Calculating the coefficient of regression, the OR value was calculated by SPSS data transformation. All statistical analyses were performed using SPSS version 25.0 software (SPSS Inc. Chicago, IL, United States).

## 3. Results

### 3.1. Patient characteristics

Patient characteristics: During the study period, 279 patients underwent PD with the same procedure by the same surgical team. The cases from 19 subjects were excluded from the analysis due to insufficient medical record, and the final index population was composed of 260 subjects. 62 PD were performed for pancreatic tumor, 109 patients underwent PD for bile duct tumor, 90 patients underwent PD for Duodenal tumor, and 3 patients underwent PD for others. The details of these patients are represented in Table 1. Among the continuous variables, only albumin was in normal distribution (average ± standard deviation), and the rest were expressed as median [interquartile range].

**Table 1:** Patient characteristics (*n* = 260).

Variable	*
Age (years, range 33–82)	59 [51–64]
**Gender**	
Female	107 (41.2)
Male	153 (58.8)
Height (cm)	160 [156–165]
Weight (kg)	57 [51–62]
BMI (kg/m^2^)	22.2 [20.2–24.2]
Underweight (≤18.5)	22 (8.5)
Normal (18.5–25)	197 (75.8)
Overweight (25–30)	37 (14.2)
Obese (≥30)	4 (1.5)
**Comorbidities**	
Hypertension	45 (17.3)
Diabetes	30 (11.5)
**Laboratory value**	
Hemoglobin (g/dl)	12.7 [11.3–13.88]
Total bilirubin (μmol/L)	140.7 [29.2–229.3]
Albumin (g/dl)	4.1 ± 0.4
**Diagnoses**	
Pancreatic tumor	62 (23.8)
Bile duct tumor	105 (40.4)
Duodenal tumor	90 (34.6)
Others	3(1.2)

*Data are shown as mean ± SD, median [interquartile range] or absolute *n* (%). BMI, body mass index.

### 3.2. Univariate and multivariate logistic analyses

The age, sex, BMI, comorbidities (hypertension, diabetes), and laboratory value (hemoglobin, total bilirubin and albumin) were used to evaluate the occurrence of Clavien-Dindo Score ≥3b after operation in univariate logistic regression analysis. Age (OR = 1.09, *P* = 0.002), hemoglobin (OR = 0.86, *P* = 0.056) and albumin (OR = 0.34, *P* = 0.014) were significantly correlated with postoperative complications. Multivariable analyses were carried out in age, hemoglobin, albumin, and patients who evaluated Clavien-Dindo Score ≥3b. Only Age (OR = 1.08, *P* = 0.01) was significantly correlated with postoperative complications (Table 2).

**Table 2:** Univariate and multivariate analyses of preoperative predictors associated with the occurrence of Clavien-Dindo Score ≥3b.

Pre-operative variable	Univariate analysis			Multivariate analysis		
	OR	95% CI	P-value	OR	95% CI	P-value
Age (years)	1.09	1.04–1.15	**<0.001**	1.08	1.03–1.14	**0**.**001**
Male	0.73	0.32–1.63	0.440			
BMI (kg/m^2^)	1.01	0.89–1.12	0.863			
Comorbidities						
Hypertension	0.50	0.21–1.21	0.126			
Diabetes	1.15	0.32–4.04	0.831			
Laboratory value						
Hemoglobin (g/dl)	0.86	0.74–1.00	**0**.**056**	0.90	0.75–1.08	0.239
Total bilirubin(μmol/L)	1.00	1	0.930			
Albumin (g/dl)	0.34	0.14–0.80	**0**.**014**	0.54	0.20–1.44	0.218

BMI, body mass index.

Bold values indicates the P value of potential key indicators.

### 3.3. ROC curve analysis

**Figure 2:**
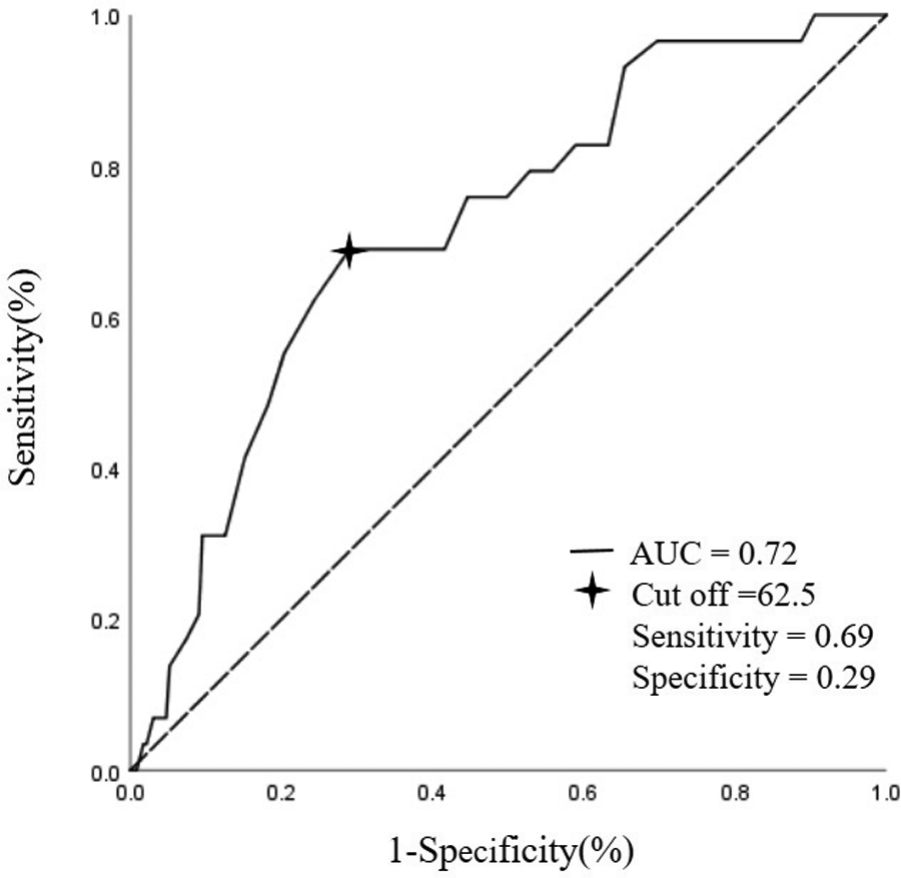
Age correlated with prediction of clavien-dindo score ≥3b in ROC curve analysis. AUC, area under curve.

At ROC curve analysis, age correlated with prediction of Clavien-Dindo Score ≥3b (AUC of 0.71, 95%CI 0.62–0.81, *P* < 0.001).Point-by point analysis of the ROC curve identified 62.5 years as the cut-off value maximizing the balance of sensitivity and specificity in the prediction of Clavien-Dindo Score ≥3b (Figure 2). According to the cut-off point of 62.5 years old, patients were divided into two groups: group I (<62.5 years) and group II (≥62.5 years). The short-term outcomes (complications, reoperation perioperative deceases, readmission, hospital stay, and cost) were analyzed in Table 3.

**Table 3:** Short-term outcomes of two groups who had undergone pancreaticoduodenectomy.

Pre-operative variable	Group I	Group II	*P*-value
	(173 pts < 62.5 yrs)	(87 pts ≥ 62.5 yrs)	
Clavien-Dindo Score ≥3b, *n* (%)	9 (5.2)	20 (23.0)	**<0.001**
Biliary leak, *n* (%)	5 (2.9)	2 (2.3)	1
POPF, *n* (%)	12 (6.9)	13 (14.9)	**0**.**039**
Chyle leak, *n* (%)	7 (4.0)	3 (3.4)	1
Postoperative bleeding, *n* (%)	15 (8.7)	7 (8.0)	0.864
Intraperitoneal infection, *n* (%)	20 (11.6)	11 (12.6)	0.799
Delayed gastric emptying, *n* (%)	11 (6.4)	10 (11.5)	0.152
Pulmonary infection, *n* (%)	17 (9.8)	7 (8.0)	0.640
Incision dehiscence, *n* (%)	0	0	1
Pleural effusion, *n* (%)	45 (26.0)	25 (28.7)	0.640
Ascites, *n* (%)	56 (32.4)	26 (29.9)	0.684
Wound infection, *n* (%)	20 (11.6)	5 (5.7)	0.134
Reoperation, *n* (%)	5 (2.9)	2 (2.3)	0.781
Perioperative deceases, *n* (%)	3 (1.7)	8 (9.2)	**0**.**013**
Readmission, *n* (%)	29 (16.8)	10 (25.6)	0.262
Hospital stay (days)	15 [12–21]	16 [13–22]	0.332
Cost (¥)	77,753.2 [58,554.4–94,430.9]	82,518.5 [60,284.8–105,890.3]	0.162

Data are shown as median [interquartile range] or absolute *n* (%).

Perioperative deceases: death within 30 days of surgery or during the index hospitalization. POPF, Postoperative pancreatic fistula; Hospital stay, length of stay after operation.

Bold values indicates the P value of potential key indicators.

### 3.4. Short-term outcomes of two groups

There were 173 (66.5%) patients in group I and 87 (33.5%) in group II. Complications are reported in Table 3: no significant differences were reported for biliary leak, postoperative bleeding, intraperitoneal infection, delayed gastric emptying, pulmonary infection, incision dehiscence, pleural effusion, ascites, wound infection, reoperation, readmission hospital stay, and cost. Perioperative deceases were 11 (4.2%), including 8 (9.2%) from among the group II (*P* = 0.013). Rates of complications with grade ≥3b, according to the Clavien-Dindo Score, were significantly different between the two groups, as well as incidence of POPF (6.9% in group I vs. 14.9% in group II; *P* = 0.039).

### 3.5. Ordinal logistic analysis

The age, hemoglobin and albumin that had influence on Clavien-Dindo ≥3b status were included in the preoperative variables, and then the ordinal logistic analysis was used to analyze the influence on the grade of Clavien-Dindo Score (Table 4). It can be seen that age (OR = 1.02, *P* = 0.085), hemoglobin (OR = 1.00, *P* = 0.936) and albumin (OR = 1.32, *P* = 0.378) have no significant effect on the level of Clavien-Dindo Score.

**Table 4:** Ordinal logistic analysis of predictors of Clavien-Dindo score in patients who underwent PD.

Variable	OR	95% CI	*P*-value
Age (years)	1.02	1.00–1.05	0.085
Hemoglobin (g/dl)	1.00	0.90–1.13	0.936
Albumin (g/dl)	1.32	0.71–2.44	0.378

## 4. Discussion

The world's population is aging rapidly. In parallel, the rate of neoplastic diseases and the number of surgical operations is rising steeply. Despite the poor outcomes, PD is the best choice for the treatment of periampullary neoplasms. The high risk of outcomes are mainly owing to the high postoperative morbidity and mortality rates. Consequently, careful selection before surgery is the key to excellent outcomes. The top factor which attracts the attention of surgeons was age, and the advanced age of patients tends to be closely related to higher postoperative complications. However, the definition of the “old patient” in terms of age varies across the studies is not worldwide accepted and different age cut-off, such as 65, 70, 75, and 80 years have been used in the studies of PD (3, 16).There is a lack of age grouping based on postoperative complications of PD. As an operation with huge trauma, defining the cut-off of elderly patients in PD can be conducive to predicting poor prognosis.

In the present study, we calculated the influence of preoperative and pathological data of 260 PD patients on Clavien-Dindo ≥3b status. In univariate logistic regression analysis, age, hemoglobin and albumin had significant effect on Clavien-Dindo ≥3b status. Age (OR = 1.09, *P* = 0.002) is a risk factor for postoperative complications, while hemoglobin (OR = 0.86, *P* = 0.056) and albumin (OR = 0.34, *P* = 0.014) are protective factors. Nutritional status before surgery is regarded as one of the key factors that influence outcomes after operation, several studies on PD have indicated the importance of maintaining normal preoperative albumin levels (17, 18). Subsequent multivariate logistic regression analysis showed that age was significantly correlated with prognosis (OR = 1.08, *P* = 0.01). Consistent with other studies, old age is an independent risk factor for complications after PD, but there is no clear cut-off point for the division of age. At ROC curve analysis, age correlated with Clavien-Dindo ≥3b status (AUC = 0.72, cut-off = 62.5). Sensitivity, and specificity of possible cut-offs for age are displayed in Figure 2. This is different from the cut-off value of aging in developed countries (65 years), which may be due to the consumption of body by malignant tumor and the larger injury of PD (19–21).

Although elderly patients were not the absolute contraindication of PD, the incidence rate of postoperative complications was higher in elderly patients. In our research, the patients were divided into two groups according to 62.5 years old: group I (<62.5 years) and group II (≥62.5 years). The short-term outcomes (complications, perioperative deceases, readmission, hospital stay, and cost) were analyzed. Among the complications, only Clavien-Dindo >3b status (5.2% in group I vs. 23.0% in group II; *P* < 0.001) and POPF (6.9% in group I vs. 14.9% in group II; *P* = 0.039) had significant differences between the two groups, and the incidence rate of the second group was significantly higher than that of the first group. The Clavien-Dindo grade and POPF showed the same trend because the main complication requiring intervention after PD was pancreatic fistula and the pancreatic anastomosis carries the highest risk of leak and cause of morbidity and mortality (22, 23). Elderly patients were prone to POPF for the following reasons: firstly, elderly patients were complicated with more basic diseases, and anastomotic edema was vulnerable to cause POPF; Second, postoperative infection increased the incidence of pancreatic fistula because of poor immunity; Third, weak physical conditions were often not conducive to the recovery of anastomotic stoma, resulting in anastomotic failure. Due to the high incidence rate of POPF and the weakness of the elderly, the perioperative mortality of the group I was significantly higher than the group II (1.7% in group I vs. 9.2% in group II; *P* = 0.013).

In terms of postoperative management, there were no significant difference in reoperation readmission, hospital stay and cost. The ordinal logistic analysis was used to analyze age (OR = 1.02, *P* = 0.085), hemoglobin (OR = 1.00, *P* = 0.936) and albumin (OR = 1.32, *P* = 0.378) on the grade of Clavien-Dindo Score, while all of them had no significant effect on this grade. We speculate that the number of high-grade of Clavien-Dindo Score is less leading to unclear judgment.

With the development of surgical technology and neoadjuvant therapy, the elderly have no longer been the forbidden area of PD surgery (7, 24, 25). However, compared with the young, the postoperative complications and mortality of the elderly have significantly increased (26, 27). Elderly patients were more likely to develop POPF and Clavien-Dindo Score ≥3b than younger ones, probably as a result of the poor recovery of pancreaticojejunostomy, frailty, and the decrease of physical tolerance in the elderly (28–30). Our research further supports the above view.

The difference is that the age group used in our study is not the commonly used 65 year old age limit in developed countries, or 75 and 80 years old in other PD prognosis studies, but the best cut-off value of 62.5 years old selected after calculating the dimensional cut-off maximizing the balance between sensitivity and specificity by analyzing the characteristics of PD patients in our hospital. Surgeons can refer to this cut-off value before making surgical decisions, and be vigilant for elderly patients to reduce postoperative complications.

The main limitation of this study is the retrospective nature and the resulting selection bias, because the cases extracted from our center were used to represent the analyzed population. Additionally, due to the difficulty of long-term follow-up, we cannot collect the long-term outcomes after PD. Therefore, further multicenter prospective studies or basic research will be needed to understand the influence of age on the short-term and long-term outcomes of PD patients.

In conclusions, age, hemoglobin and albumin were significantly correlated with postoperative Clavien-Dindo Score ≥3b in preoperative variables. According to 260 cases in our center, the cut-off value of elderly patients with PD was 62.5 years old and there were significant differences in postoperative Clavien-Dindo Score ≥3b, pancreatic fistula, and perioperative death between the elderly and young group. However, age, hemoglobin and albumin were no significant differences in predicting the grade of Clavien-Dindo Score.

## Data Availability

The original contributions presented in the study are included in the article/Supplementary Material, further inquiries can be directed to the corresponding author.
